# Gas chromatography tandem mass spectrometry offers advantages for urinary steroids analysis

**DOI:** 10.1016/j.ab.2017.09.002

**Published:** 2017-12-01

**Authors:** Natalie Homer, Sanjay Kothiya, Alison Rutter, Brian R. Walker, Ruth Andrew

**Affiliations:** a Edinburgh Clinical Research Facility Mass Spectrometry Core, Queen's Medical Research Institute, University of Edinburgh, 47, Little France Crescent, Edinburgh, EH16 2TJ, UK; b University/British Heart Foundation Centre for Cardiovascular Science, Queen's Medical Research Institute, University of Edinburgh, 47, Little France Crescent, Edinburgh, EH16 2TJ, UK

**Keywords:** Steroid, Triple quadrupole, Tandem mass spectrometry, Urine, Corticosteroid, Androgen

## Abstract

Gas chromatography mass spectrometry has been the lynchpin of clinical assessment of steroid profiles for ∼3 decades. The improvements in assay performance offered by tandem mass spectrometry were assessed. Across the spectrum of glucocorticoid and androgen analytes tested, limits of detection and quantitation were ∼20 fold lower with triple than single quadrupole systems, but the more noticeable improvement was that signal to noise was substantially improved and the linear range wider. These benefits allowed more reliable and concomitant measurement of steroids with substantially different abundances and in smaller volumes of urine.

## Introductory statement

Profiling of urinary metabolites of adrenal and gonadal steroids has been performed routinely for approximately 30 years, with several seminal papers by Shackleton et al. [1], [2] defining an approach that is used worldwide in research and clinical diagnosis, also being extended to the fields of toxicology and doping. Clinically, such analysis has been used widely in the diagnosis of inborn errors of metabolism and more recently in the differential diagnosis of adrenal tumours [3]. Originally a profile of steroids measured as their methoxyamine-trimethylsilyl derivatives was performed by gas chromatography (GC) with detectors such as Flame Ionisation detectors (FID), but more recently steroids have been identified by single quadrupole mass spectrometers (MS). The use of MS enhances specificity over FID, but challenges remain. The amounts of steroids and their metabolites differ greatly both between analytes and disease states, meaning calibration ranges can vary substantially. In particular some steroids are present in low abundance (e.g. cortisol and cortisone) and close to the acceptable limits for quantitation (LOQs), even following extraction of relatively large volumes (∼20 mL) of urine.

Signal to noise can be improved using tandem MS (MS/MS), lowering the LOQs. Recently there has been a move towards liquid chromatography (LC)-MS/MS as the method of choice for quantitative analysis. Higher throughput can be achieved as derivatisation is not routinely required. Despite poor ionisation efficiency, some neutral steroids can be successfully quantified by electrospray (ESI) and atmospheric pressure chemical (APCI) ionisation, although their signal is susceptible to ion suppression by the matrix. Nonetheless the more abundant circulating pregnene and androstene steroids, such as cortisol and testosterone, are now routinely quantified by this means [4]. However their metabolic products, some of which retain biological activity [5], cannot be detected nor discriminated readily using LC-MS/MS. Metabolism of the keto-enol function in the A-ring forms 5α and 5β-reduced dihydro products, followed by subsequent reduction of the 3-keto by 3α and β-hydroxysteroid dehydrogenases [6] and yields metabolites that ionise poorly. Moreover separation of multiple sets of stereo-isomers is problematic by LC.

GC-MS/MS therefore holds inherent advantages for steroid hormones as they present a portfolio of lipophilic analytes with close structural similarity. The isomeric derivatives formed for GC analysis can be resolved more readily, facilitating stereochemical discrimination. Furthermore, the intensity of the signal from the derivatives of the metabolites is not compromised compared to the precursor hormone. We have adapted the GC-based approach of urinary steroid analysis by single quadrupole analysis to exploit the power of tandem mass spectrometry and report here the validated assay.

## Materials and methods

### Instrumentation

Analysis was performed on a Trace GC Ultra, equipped with a DB17MS column (30 m, 0.25 mm, 0.25 μm, Agilent Technologies Ltd. Stockport, UK), fitted with a AS autosampler and interfaced with a TSQ Quantum triple quadrupole MS (Thermoscientific, Hemel Hempstead, UK). Data were processed using Xcalibur Software version 3.0. Samples (1 μL) were injected (120 °C) using a Programmed Temperature Vaporising injector, with a constant helium flow of 1.5 mL/min. The injector temperature was increased to 270 °C at 14.5 °C/min, held for 1 min and then increased at the same rate to 300 °C, held for 10 min and returned to baseline. The initial GC temperature was 120 °C and increased (30 °C/min) to 200 °C, then after 1 min increased (5 °C/min) to 300 °C and sustained for 5 min. The auxiliary line and source temperatures were 280 °C. The MS was operated in electron impact (70 eV), using argon as a collision gas, at a pressure of 1 mTorr.

### Sources of chemicals

Unlabelled steroids (Table 1) and internal standards were from Steraloids (Rhode Island, USA). Other chemicals were from Sigma-Aldrich (Poole, UK) unless otherwise stated. Lipidex-5000 was from Perkin-Elmer (Cambridge, UK). Solvents were glass-distilled HPLC grade (Fisher Scientific, Loughborough, UK). Trivial and systematic names of steroid metabolites are 5β-tetrahydrocortisol (5βTHF), 3α,11β,17α,21-tetrahydroxy-5β-pregnan-20-one; 5α-tetrahydrocortisol (5αTHF), 3α,11β,17α,21-tetrahydroxy-5α-pregnan-20-one; tetrahydrocortisone (THE), 3α,17α-21-trihydroxy-5β-pregnan-11,20-dione; α-cortol, 5β-pregnane-3α,11β,17,20α,21-pentol; β-cortol, 5β-pregnane-3α,11β,17,20β,21-pentol, α-cortolone, 3α,17α,20α,21-tetrahydroxy-5β-pregnan-11-one; β-cortolone, 3α,17α,20β,21-tetrahydroxy-5β-pregnan-11-one; etiocholanolone, 5β-androstan-3α-ol-17-one; androsterone, 5α-androstan-3α-ol-17-one.

**Table 1:** Mass spectrometric and assay validation parameters for analysis of steroid derivatives by gas chromatography tandem mass spectrometry.

**Table 1A**							
Steroidal derivative	Mass Transition (*m*/*z*) Precursor → Product		Collision Energy	Replicate Injections % RSD			Aqueous Recovery
Analytes	Quantifier	Qualifier	Qn, Ql (V)	Low	High	Urine	%
(5α)-Androsterone	360 → 270	360 → 213	15,15	3.5	0.9	1.1	90.5
(5β)-Etiocholanolone	360 → 270	360 → 213	15,15	4.0	1.2	2.1	91.8
5αTHF	652 → 562	562 → 472	15,20	1.1	1.9	4.4	111.6
5βTHF	652 → 562	562 → 472	15,20	1.9	0.6	2.2	109.5
THE	578 → 398	578 → 488	15,15	2.7	0.4	0.7	112.5
α-Cortol	535 → 445	535 → 355	15,20	2.4	1.8	5.3	96.4
β-Cortol	535 → 445	535 → 355	15,20	2.4	0.7	2.7	115.7
α-Cortolone	449 → 269	449 → 359	10,15	8.9	1.1	0.6	94.1
β-Cortolone	449 → 269	449 → 359	10,15	8.0	1.2	1.5	112.3
Cortisol	605 → 515	515 → 425	20,15	11.8	2.1	4.1	104.9
Cortisone	531 → 441	441 → 382	15,15	3.7	2.0	2.8	119.5
**Internal Standards**							
11-Epi-Cortisol	605 → 515	515 → 425	20,15				94.4
11-Epi-THF	652 → 562	562 → 472	15,20				97.2
3α,17α -Androstanediol	331 → 241	331 → 255	15,10				103.1

**Table 1B**							

Extracted ion chromatogram of derivatised cortisone analysed by (i) single quadrupole or (ii) triple quadrupole mass spectrometry following gas chromatographic separation							

RSD = Relative Standard Deviation; Qn = Quantifier; Ql = Qualifier; Tetrahydrocortisol = THF, Tetrahydrocortisone = THE.

### Derivatisation of steroids or extracts

Steroids were dissolved in methanol (1 mg/mL) and stored at −20 °C. Serial methanolic dilutions were performed and aliquots of standards or whole extracts reduced to dryness under oxygen-free nitrogen (60 °C) in Reacti Vials. Methoxime-trimethylsilyl derivatives were formed [7] and dissolved in decane (200 μL) and 1 μL injected.

### MS parameters

Full scans of derivatised standards were performed in Q1 (100 ng/200 μL). Potential precursor ions were subject to collision (increments of 5 between 0 and 40 V), and product ion scans collated. Four suitable mass transitions were initially selected for dynamic selected reaction monitoring (SRM) and the two yielding the most intense signals for standards and subsequently best signal to noise in representative biological samples selected as quantifier and qualifier ions (Table 1). Time windows of 3 min were typical. Steroids co-eluting (e.g. analyte and stable-isotope labelled internal standard) were examined separately to ensure lack of interference between ion transitions.

### Extraction of urinary steroids

Steroids were extracted from aliquots (1-20 mL) of 24 h urine collected from healthy male subjects [8] with ethical approval and pooled into 6 replicates (n = 6). Water was used as the matrix for calibration standards.

### Assay validation

Limits of detection (LODs) of unextracted steroid standards were assigned as peaks with signal-to-noise of >3. Recovery of analytes was assessed from water by comparing signal intensity after extraction with matched unextracted standards. Calibration curves were prepared with serial dilutions of analytes (lowest point LOD) and fixed quantities of internal standards (11-epi-cortisol (1.5 μg), 11-epi-tetrahydrocortisol (15 μg), androstandiol (5 μg). The following amounts were included in the calibration curves: 5βTHF, 5αTHF, THE (0.05, 0.1, 0.5, 1, 2, 5*, 10, 20, 30, 40, 50, 100, 150, 200, 300^#^ μg); androsterone, etiocholanolone, α and β-cortol, α and β-cortolone, cortisol and cortisone (0.025, 0.05, 0.25, 0.5, 1, 2.5*, 5, 10, 15, 20, 25, 50, 75, 100, 150^#^ μg). Linearity was assessed by least squared regression analysis and weightings (equal, 1/x and 1/x^2^) compared. The precision of replicate injections was assessed by reinjecting the same standard (low* being either 2.5 or 5 μg, high^#^ being either 150 or 300 μg) 6 times. In 6 replicates of standards of the amounts equivalent to the lower limit of quantitation and high amounts as above, accuracy (relative mean error; RME) and precision (% relative standard deviation; RSD) were assessed following extraction (intra- and inter-assay). The limits of quantitation (LOQs) were assigned where accuracy and precision achieved %RME and %RSD <20%. At other points, values <15% were deemed acceptable. (RME= (Amount measured-theoretical amount)/theoretical amount; RSD = Standard Deviation of replicates/Mean).

Recovery of internal standards from urine was assessed by comparing results of samples spiked before and after extraction. The precision of replicate injections of urine (1 or 10 mL) was assessed by reinjecting the same sample 6 times. Replicates (n = 6) of pooled urine sample were analysed on the same day and reanalysed after 12 months storage at −20 °C and precision of analysis assessed.

## Results and discussion

The existing method for single quadrupole analysis was adapted for triple quadrupole detection. Ions previously selected for single quadrupole analysis were uniformly present in the Q1 scans, and ultimately selected as the precursor ions for SRM. The transitions (Table 1) yielding the most intense products ions, with the greatest signal to noise ratios were selected and largely represented loss of derivative functions (*m/z* minus 90 or 31) as predicted from full scan spectra. Collision energies were selected to retain small amounts of precursor ion (<5% where possible). Raising the source temperature noticeably improved signal intensity (280 vs 200 °C), possibly reflecting manufacturer-specific source design. Chromatographic conditions required adjustment potentially to accommodate interfacing with a PTV injection as opposed to split-splitless injection mode, PTV offering advantages with cleaner sample introduction.

### Linear range of quantitation

Replicate injections of standards yielded peak areas recorded with RSDs of 1.1–11.8% (Table 1). The LODs of unextracted standards was approximately 500 pg on column for all steroids. Broadly the LOQ was 10–20 fold fold higher than the LOD and this increment was most marked for metabolites with poorer chemical similarity to the internal standard used. Quantitation was linear over a wider dynamic range than observed previously using single quadrupole analysis. Although not routinely required, 3 orders of magnitude dynamic range could be achieved, if higher concentration samples were diluted to volumes larger than 200 μL. Generally, a weighting of 1 yielded best fit lines and good fit of the low points was better achieved by limiting the range of the line to one order of magnitude, rather than by adjusting weighting.

Recovery of standards and internal standards from water was excellent and close to 100% (Table 1). Intra- and inter-assay precision and accuracy of extracted standards are in Table 2.

**Table 2:** Lower and upper limits of quantitation (LLOQ/ULOQ) of steroidspre-and post-extraction.

	LLOQ Standards Extracted (n=6)					ULOQ Standards Extracted (n=6)					Urine (n=6)			
		Intra-assay		Inter-assay			Intra-assay		Inter-assay		Intra-assay Precision (%)			% Original
Steroids	LLOQ ng on column	%RSD	%RME	%RSD	%RME	ULOQ ng on column	%RSD	%RME	%RSD	%RME	IS Post-spiked	IS Pre- Spiked 10 mL	IS Pre- Spiked 1 mL	20 °C, 12 months
Androsterone	12.5	4.9	112.8	17.1	103.9	750	7.8	95.7	13.5	96.05	5.2	4.5	11.7	109.2
Etiocholanolone	12.5	7.7	113.0	8.3	100.8	750	8.9	111.4	3.4	99.95	5.7	4.5	10.6	105.3
5βTHF	25	13.5	95.7	7.9	102.7	1500	2.5	107.6	2.7	101.2	6.5	2.0	3.2	93.0
5αTHF	25	7.5	94.7	12.9	107.7	1500	3.5	101.5	2.4	101.2	6.3	1.9	6.4	80.0
THE	25	8.6	94.0	14.8	105.4	1500	4.2	88.7	1.9	101.7	6.5	1.6	0.9	100.8
α-cortol	12.5	11.8	100.8	6.0	89.3	750	4.5	86.7	1.4	102.7	10.8	6.5	8.7	102.1
β-cortol	12.5	12.9	105.4	20.2	86.3	750	3.5	98.8	2.8	101.8	5.4	7.5	6.4	109.8
α-cortolone	12.5	15.5	113.4	20.6	90.7	750	3.1	99.4	4.9	100.5	8.5	3.0	5.4	115.2
β-cortolone	12.5	13.4	106.2	12.8	106.0	750	2.7	99.1	2.6	99.6	8.0	6.4	5.8	97.2
cortisol	2.5	6.2	102.6	10.3	95.0	150	2.4	99.5	1.8	100.5	6.3	4.3	7.5	109.2
cortisone	2.5	14.0	100.2	12.2	102.7	150	2.8	99.6	1.0	101.6	7.1	3.4	3.9	105.3

RSD = Relative Standard Deviation; RME = Relative Mean Error; IS = internal standard; L/ULOQ lower and upper limit of quantitation.

### Application to biological samples

Recovery of internal standards from urine samples was ∼66–72% and the extraction process did not substantially influence the variability of recovery as seen by comparing RSD pre- or post-spiking the internal standard (Table 2). Replicate injections of urine samples yielded precise results, comparable to standards (Table 1). Precision was improved in urinary extraction by reducing the amount of sample per Sep-pak. Protocols have been reported that employ the use of the same cartridge for the initial extraction and the extraction post-hydrolysis [2]. This was acceptable when volumes of up to 10 mL were extracted but proved less reliable at larger volumes for 300 mg cartridges. Excellent precision was also sustained when volumes were scaled down to 1 mL with all analytes detectable, not previously achievable with single quadrupole systems. This has advantages in speed and cleanliness for instrument maintenance and is potentially scalable to a 96 well plate format.

Signal to noise ratios of analytes in biological matrix measured by triple quadrupole MS were far superior to single quadrupole analysis. Exemplary mass chromatograms are shown in Table 1B with much reduced background noise. Intra-assay precision was excellent (Table 2). Subsequent re-injection of derivatised samples after storage at −20 °C for 12 months showed that the signal for analytes was stable during this period, with declines in reported abundance of no greater than 20%.

## Conclusions

Triple quadrupole MS offers advantages over conventional single quadrupole analysis and advances the performance of urinary steroid profiling. The most notable improvements were in the signal to noise as well as the dynamic range. This was reflected in better performance of the urinary steroid assay in terms of precision and accuracy compared with our previous single quadrupole method. For higher abundance analytes analysis of volumes of 1 mL may be performed but there may still advantages to processing larger volumes to increase the scope of the metabolite profile or record quantities of unconjugated steroids. This approach will take its place among other MS techniques and may be compared by other contemporary approaches being explored at the GC interface such as convergence chromatography [9] which allows more rapid chromatography and higher throughput.
